# The Impact of Digital Therapeutics on Current Health Technology Assessment Frameworks

**DOI:** 10.3389/fdgth.2021.667016

**Published:** 2021-06-09

**Authors:** Kevin Yan, Chakrapani Balijepalli, Eric Druyts

**Affiliations:** ^1^Pharmalytics Group, Vancouver, BC, Canada; ^2^Faculty of Medicine, University of British Columbia, Vancouver, BC, Canada

**Keywords:** digital therapeutics, digital health, digital health technology, health technology assessment, reimbursement

## Abstract

Historically healthcare has been delivered offline (e.g., physician consultations, mental health counseling services). It is widely understood that healthcare lags behind other industries (e.g., financial, transportation) whom have already incorporated digital technologies in their workflow. However, this is changing with the recent emergence of digital therapeutics (DTx) helping to bring healthcare services online. To promote adoption, healthcare providers need to be educated regarding the digital therapy to allow for proper prescribing. But of equal importance is affordability and many countries rely on reimbursement support from the government and insurance agencies. Here we briefly explore how national reimbursement agencies or non-profits across six countries (Canada, United States of America, United Kingdom, Germany, France, Australia) handle DTx submissions and describe the potential impact of digital therapeutics on current health technology assessment (HTA) frameworks. A targeted review to identify HTA submissions and guidelines from national reimbursement agencies or non-profits was conducted. We reviewed guidelines from the Institute for Clinical and Economic Review (ICER) in the USA, the Canadian Agency for Drugs and Technologies in Health (CADTH) in Canada, the National Institute for Health and Care Excellence (NICE) in the United Kingdom (UK), the Institute for Quality and Efficiency in Health Care (IQWIG) in Germany, Haute Autorité de Santé (HAS) in France, and the Pharmaceutical Benefits Advisory Committee (PBAC) in Australia. Our review identified one set of guidelines developed by NICE in the UK. The guidelines by NICE outlined an evidence standards framework for digital health technologies (DHT). Depending on the organizational impact, financial commitment, and economic risk for the payer, different economic analyses are required. Economic analyses levels are separated into 3 categories, basic, low financial commitment, and high financial commitment. All economic analyses levels require a budget impact analysis. A cost-utility analysis is recommended for DHTs categorized in the high financial commitment category. Whereas, for DHTs that are in the low financial commitment category, a cost-consequence analysis is typically recommended. No HTA guidelines for DTx submissions were identified for the remaining countries (Canada, USA, Germany, France, and Australia)

## Background

The majority of healthcare is currently delivered offline (e.g., physician consultations, pharmacy prescriptions, mental health counseling services). It is widely understood that healthcare lags behind other industries (e.g., financial, transportation) which have already incorporated digital technologies in their workflow (1). However, this is changing with the recent emergence of digital therapeutics (DTx) helping to bring healthcare services online. Digital therapeutics can be defined as a regulatory approved digital system or application that is prescribed to treat medical conditions, similar to that of new drug molecules or medical devices (2–6). As developers of digital therapeutics pass regulatory approval, the next step is to gain widespread adoption. To promote adoption, healthcare providers need to be educated regarding the digital therapy to allow for proper prescribing. But also, of equal importance is patient affordability of which a key determinant in many countries relies on reimbursement support from the government and insurance agencies. Transition to incorporate digital technologies into clinical practice have been slow due to strict regulations and the disparity between stakeholder views of this new change (7). If proven effective, with the introduction to virtual care, it will lead to great advances in convenience, accessibility, and potentially better outcomes for patients (8, 9). Moreover, it will allow for healthcare providers to conveniently monitor, educate, and adjust therapeutic regimens for an increased number of patients (8, 10). However, this will not be without challenges, including the need to prove value compared to current interventions and demonstrate potential cost savings for payers.

There has been doubt to whether cost savings can truly be achieved when using DTx (11). Experts have argued that if these DTx were proven to be cost-effective, it would likely result in a net overall increase in healthcare spending (12). Governments are exploring to introduce additional billing codes to support digital care monitoring which can potentially lead to increased spending (9). Moving forward, as a DTx software evolves when incorporating more data, this may impact current and future HTA submission recommendations. Here we explore how health technology assessment (HTA) agencies handle DTx submissions. We conducted a targeted review to identify HTA submissions and guidelines from national reimbursement agencies or non-profits across six countries (Canada, USA, United Kingdom, Germany, France, Australia). The following agencies were reviewed: the Institute for Clinical and Economic Review (ICER) in the USA, the Canadian Agency for Drugs and Technologies in Health (CADTH) in Canada, the National Institute for Health and Care Excellence (NICE) in the United Kingdom (UK), the Institute for Quality and Efficiency in Health Care (IQWIG) in Germany, Haute Autorité de Santé (HAS) in France, and the Pharmaceutical Benefits Advisory Committee (PBAC) in Australia.

## Pharmaceutical Drugs Vs. Digital Therapeutics

Unlike pharmaceuticals whereby a drug is administered into the body, a DTx relies on extraneous factors such as a stable internet connection, complimentary cellular device, or proficient user interaction. When conducting a health technology assessment and determining parameters such as epidemiology estimates of the disease and the associated economic costs for implementing a DTx intervention, a stakeholder must consider if the end user (i.e., patient) has the adequate technology and/or user ability to operate the technology. Due to the nature of DTx, only patients who can operate and afford technological hardware may benefit leading to potential bias. Studies have shown individuals of higher socioeconomic and education status tend to be healthier and have healthier behaviors (13–15). Age is also an important factor as older individuals may not own or be familiar using smart technologies (i.e., smartphones, tablets) (16). Moreover, other identified barriers for older individuals include the complexity and lack of guidance when using these technologies (16). The elderly may also not be interested in learning how to use new DTx interventions even if this could potentially be the population that would receive the largest benefit (17). These older individuals would not need to physically visit their physician's office for every appointment and could use remote monitoring as a tool to improve overall health.

Age and socioeconomic factors need to be examined (e.g., education level, family income) as these digital interventions require the necessary user ability and hardware to function. Thus, when DTx are indirectly compared to pharmacologic treatments for HTA purposes, baseline characteristics may potentially need to be adjusted more heavily for socioeconomic factors. Unlike pharmacological drugs whereby it impacts a biological mechanism (e.g., SGLT2 inhibitor for diabetes or antiplatelet agents to prevent cardiovascular events), DTx heavily relies on an individual's behavior and attitudes toward health. Contrary to pharmaceutical drugs whereby consideration for human biological factors is necessary to assess for potential therapeutic effectiveness (e.g., ancestry, genetics), in digital therapeutics technology literacy, age and other socioeconomic factors (e.g., income, nationality) may potentially play a larger role when conducting reimbursement decisions.

As DTx is implemented and evolve with capabilities, will payers be expected to pay for initial and future training and operation costs associated with these technologies? Moreover, as the technology accumulates increasingly more data, the evidence will also need to be consistently updated. Assuming the technology evolves, this may impact prior HTA results, potentially making the intervention more or less cost-effective. In contrast, with pharmaceutical drugs, the effectiveness of the drug does not change. A potential solution would be incorporating dynamic HTAs whereby the evidence is consistently updated throughout predetermined time intervals as a part of post-market surveillance. However, challenges to this include accurately isolating therapeutic effectiveness as a result of the intervention's technology or other associated factors.

## Digital Therapeutics and Health Technology Assessment

Our review identified one set of guidelines developed by NICE in the UK (18). NICE has outlined an evidence standards framework for digital health technologies (DHT) and has compartmentalized DHTs into functional groups. These functional classes are grouped into evidence tiers and are intended to capture the level of clinical risk associated with the DHT. There are three evidence tiers; Tier A: system impact, Tier B: understanding and communicating, and Tier C: interventions. Tier A includes DHTs which focus on system services and have no measurable impact on patient outcomes (ex. electronic prescribing systems). Tier B DHTs focus on information, communication, and simple monitoring (ex. cognitive behavioral programmes, healthy lifestyle applications). Tier C DHTs focuses on diagnosis, treatment, and active monitoring. Examples of Tier C technologies may include DHTs that use data to assist in disease diagnosis or DHTs for treating and monitoring chronic conditions (e.g., diabetes).

Depending on the organizational impact, financial commitment, and economic risk for the payer, different economic analyses are required. Economic analyses levels are separated into 3 categories, basic, low financial commitment, and high financial commitment. All economic analyses levels require a budget impact analysis. A cost-utility analysis is recommended for DHTs categorized in the high financial commitment category. These include DHTs that obtain funding by the government for health and non-health outcomes. A cost- consequence analysis can also be conducted if evidence is not sufficient for conducting a cost-utility analysis, however, this appears to be evaluated on a case by case basis. Cost consequence analysis are also a minimum requirement for DHTs that are in the low financial commitment category (18). These evidence requirements are only directed toward digital technologies seeking public reimbursement and do not apply to unpaid interventions. It is unfortunate that no other HTA agencies we reviewed have as of yet outlined any public recommendations to guide digital health technology submissions as many DTxs have already been approved by multiple regulatory agencies. Reports and case studies evaluating existing DTx have been conducted by NICE and CADTH (19, 20). It appears the assessments and evaluations that have been conducted attempt to fit the mold of existing frameworks for pharmaceutical and medical devices. However, DTx is unique in that it is unlike traditional drugs and devices.

The issue of potential changes in effectiveness due to DTx software updates and how that will impact previous HTA assessments requires clarification. Assuming changes in effectiveness, will companies be expected to alter pricing according to local willingness to pay thresholds? A potential solution can be requiring original manufacturer model submissions to include larger variances in their sensitivity analysis, thereby accounting for a wider fluctuation in input parameters. Non-adherence to DTx needs to also be accounted for in economic evaluations and its potential impact on costs and effectiveness. Non-adherence to traditional pharmaceutical drugs is already a common issue contributing costs upwards of $50,000 per patient per year (21). Compounding the complexity from simply taking a pill to be added or replaced with navigating through a smartphone application, meanwhile, answering questions and communicating results with healthcare practitioners will most likely lead to a greater depreciation in adherence.

Traditionally, patients are prescribed and dispensed medication without knowledge of their adherence history. Revoking reimbursement privileges due to non-adherence is an ethical issue and patients should not be unnecessarily penalized for occasionally being non-adherent when they could possibly be overwhelmed with other parts of their life. Due to the technological nature and potential ability to track software usage, it is possible to restructure contemporary reimbursement strategies. Reimbursement agencies can potentially pay per active DTx use whereby the patient successfully finishes the instructions provided by the physician. In this scenario in events that the patient is non-adherent, it does not penalize the patient nor the reimbursing party. Moreover, this can complement traditional healthcare whereby non-adherent patients can be easily identified and early alternative interventions can be discussed to promote more personalized healthcare services. Nonetheless, when establishing criteria for linking reimbursement to adherence, it can create both opportunities and challenges for decision makers.

If tracking health outcomes can also be possible in real time, is it also possible that payers require certain incremental levels of health benefit for continuing reimbursement support? Similar to pharmacological therapy, patients are switched to alternative drug therapies if the initial treatment did not demonstrate adequate effectiveness (e.g., blood glucose and A1C levels in diabetes patients). However, the criteria for health benefit will need to be adequately defined to prevent removing potential patients that are benefitting from DTx. The dearth of evidence, especially high-quality evidence, associated with determining effectiveness of these interventions will also be an issue (22). If HTA agencies consider evidence as a pillar for reimbursement, DTx will not be able to compete against traditional pharmaceutical drugs with its larger evidence base. Traditional analyses such as indirect treatment comparisons or cost-utility analysis may not always be possible due to the anticipated population heterogeneity expected from DTx users.

## Looking Forward

With the recent FDA approval of the first game-based DTx used to treat ADHD, it demonstrates the expanding scope of DTx beyond patient monitoring (23, 24). Some areas of healthcare may never be fully replaced by technology, thereby, it will be likely that DTx will complement existing interventions to provide improved outcomes to patients. As DTx evolves, new HTA strategies and methods for assessing these interventions will be needed. ICER is in the process of conducting the first HTA review aimed to evaluate the health and economic outcomes of DTx in addition to medication assisted treatment in opioid use disorder (25). Based on the recent ICER protocol documents of opioid apps it appears there is a shift toward focusing on non-health related and societal based outcomes (e.g., accidental pediatric exposure, employment-related outcomes, housing-related outcomes, and relationship-related outcomes) (26). Traditional HTA methods and guidelines will need to be updated and revised to take into consideration technological and socioeconomic factors that comes with using these new technologies.

## Author Contributions

KY performed conceptualization, drafted the article, and editing. ED and CB performed supervision, review, and editing. All authors have reviewed, added value, and signed off on the final version of this manuscript.

## Conflict of Interest

The authors declare that the research was conducted in the absence of any commercial or financial relationships that could be construed as a potential conflict of interest.
